# Patterns of drug resistance among newly diagnosed HIV-1 infected patients in Greece during the last decade: the crucial role of transmission networks

**DOI:** 10.7448/IAS.17.4.19742

**Published:** 2014-11-02

**Authors:** Dimitrios Paraskevis, Assimina Zavitsanou, Emmanouil Magiorkinis, Panagiotis Gargalianos, Georgios Xylomenos, Marios Lazanas, Maria Chini, Athanasios Skoutelis, Vasileios Papastamopoulos, Anastasia Antoniadou, Antonios Papadopoulos, Mina Psichogiou, Georgios Daikos, Alexis Vassilakis, Georgios Chrysos, Vasilis Paparizos, Sofia Kourkounti, Helen Sambatakou, Theodoros Kordossis, Georgios Koratzanis, Periklis Panagopoulos, Evangelos Maltezos, Stylianos Drimis, Angelos Hatzakis

**Affiliations:** 1 Hygiene Epidemiology and Medical Statistics, Medical School, University of Athens, Athens, Greece; 2 Department of Internal Medicine, G. Gennimatas General Hospital, Athens, Greece; 3 Department of Internal Medicine, Hellenic Red Cross Hospital, Athens, Greece; 4 Department of Medicine and Infectious Diseases, Evangelismos General Hospital, Athens, Greece; 5 Department of Internal Medicine, University General Hospital Attikon, Athens, Greece; 6 Department of Propedeutic Medicine, Laiko General Hospital, Athens, Greece; 7 Department of Internal Medicine, Tzaneio General Hospital, Piraeus, Greece; 8 Department of Dermatology, A. Syngros Hospital, Athens, Greece; 9 Infectious Diseases, Ippokrateion General Hospital, Athens, Greece; 10 Department of Pathophysiology, Laiko General Hospital, Athens, Greece; 11 Infectious Diseases, Sismanogleio General Hospital, Athens, Greece; 12 Department of Internal Medicine, Democritus University of Thrace, Alexandroupolis, Greece

## Abstract

**Introduction:**

The prevalence of drug resistance is approximately 10% in Europe and North America among newly infected patients. We aim to investigate the temporal patterns of resistance among drug naive HIV-infected individuals in Greece and also to determine transmission networking among those with resistant strains.

**Materials and Methods:**

Protease (PR) and partial reverse transcriptase (RT) sequences were determined from 2499 newly diagnosed HIV-1 patients, in Greece, during 2003–2013. Genotypic drug resistance was estimated using the HIVdb: Genotypic Resistance Interpretation Algorithm. We identified transmission clusters of resistant strains on the basis of a large collection of HIV-1 sequences from 4024 seropositives in Greece. Phylodynamic analysis was performed using a Bayesian method.

**Results:**

We estimated drug resistance levels among naïve patients on the basis of all resistance mutations in PR and partial RT. The overall prevalence of resistance was 19.6% (490/2499). Resistance to NNRTIs was the most common (397/2499, 15.9%) followed by PIs (116/2499, 4.6%) and NRTIs (79/2499, 3.2%). We found a significant trend for decreasing resistance to NRTIs over time (6.7%–1.6%). There was no time trend for the overall PI and NNRTI resistance. The most frequently observed major resistant sites in PR were V82 (2.0%) and L90 (1.8%). In RT, we found E138 (58.6%), K103 (13.1%), V179 (8.4%) and T215 (7.1%), M41 (4.7%) associated with resistance to NNRTIs and NRTIs, respectively. The prevalence of K103N and E138Q were significantly increased during 2003–2013. Crucially, we found that both K103N, E138Q are associated with transmission networking within men having sex with men (MSM) and intravenous drug user (IDU) local networks. The K103N network included seropositives across Greece, while the latter only from the recent IDU outbreak in Athens metropolitan area (1). Phylodynamic analyses revealed that the exponential growth for K103N network started in 2009 (Figure 1) and for the E138Q in 2010.

**Conclusions:**

The overall resistance has been stable in Greece over time; however, specific NNRTI resistance patterns are increasing. Notably, they are associated with local transmission networking, thus suggesting that this is the cause for the increased patterns of NNRTI resistance and not multiple transmissions of resistant strains from different sources among treated individuals. Our study highlights the advance of molecular epidemiology for understanding the dynamics of resistance.

**Figure 1:**
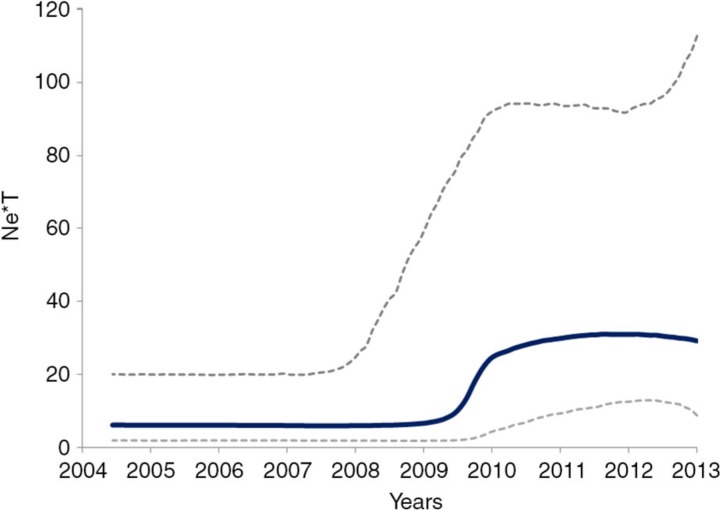
Phlodynamics of 103N.

## References

[1] Paraskevis D, Nikolopoulos G, Fotiou A, Tsiara C, Paraskeva D, Sypsa V (2013). Economic recession and emergence of an HIV-1 outbreak among drug injectors in Athens metropolitan area: a longitudinal study. PLoS One.

